# In Vitro Bioaccessibility of Edible Seaweed Proteins from the Chilean Coast and Proteins from the Novel Seaweed-Derived Mycoprotein

**DOI:** 10.3390/molecules30010165

**Published:** 2025-01-03

**Authors:** Catalina Landeta-Salgado, Javiera Munizaga, María Paz González-Troncoso, Anamaría Daza-Sanchez, Irene Martínez, María Elena Lienqueo

**Affiliations:** 1 Department of Chemical Engineering, Biotechnology, and Materials, Centre for Biotechnology and Bioengineering (CeBiB), University of Chile, Santiago 8330111, Chile; jmmunizaga@uc.cl (J.M.); maria.gonzalez.t@ug.uchile.cl (M.P.G.-T.); anitaasd@gmail.com (A.D.-S.); imartinez@ing.uchile.cl (I.M.); mlienque@uchile.cl (M.E.L.); 2 Faculty of Veterinary Medicine and Agronomy, Institute of Natural Sciences, University of the Americas (UDLA), Santiago 7500975, Chile

**Keywords:** seaweed, mycoprotein, in vitro digestibility, PDCAAS, DIAAS, amino acid, protein

## Abstract

Seaweed biomass is globally underutilized as a source of proteins despite its nutritional potential, with much of its use focused on hydrocolloid extraction. This study evaluated the nutritional quality and digestibility of protein and amino acids from two brown seaweeds (*Durvillaea* spp. and *Macrocystis pyrifera*), one green seaweed (*Ulva* spp.), and a novel mycoprotein derived from *Durvillaea* spp. through fungal fermentation. Using an in vitro gastrointestinal digestion Megazyme assay kit, protein digestibility-corrected amino acid scores (PDCAASs) and digestible indispensable amino acid scores (DIASSs) were determined. Compared with seaweeds, seaweed-derived mycoprotein presented significantly greater protein contents (~33%) and amino acid profiles (2.2 times greater than those of *Durvillaea* spp. and *M. pyrifera)*, with greater digestibility (~100%) than seaweeds (<60%). The PDCAAS values were 0.37, 0.41, 0.53, and 0.89 for *Ulva* spp., *Macrocystis pyrifera*, *Durvillaea* spp., and mycoproteins, respectively. The DIASSs highlighted the superior nutritional quality of the mycoprotein, particularly for lysine (0.59) and histidine (0.67). SDS-PAGE revealed soluble peptides (<25 kDa) in *Durvillaea* spp., *Macrocystis pyrifera*, and mycoproteins, whereas *Ulva* spp. proteins exhibited limited solubility due to structural aggregation. These findings highlight the need to characterize the nutritional properties of edible seaweeds in Chile further and emphasize the importance of optimized processing techniques, such as fermentation or bioconversion, to improve the nutritional potential of seaweeds and develop high-quality food ingredients for diverse applications.

## 1. Introduction

Current dietary habits and agricultural production methods are putting significant strain on terrestrial and aquatic ecosystems, depleting water resources, and contributing to climate change as we work to feed 7.6 billion people. Identifying solutions that can be effectively implemented across the diverse and expansive range of producers in the agricultural sector is particularly challenging [1,2]. Alternative proteins are a category of proteins obtained from non-traditional animal sources such as red meat and chicken, and they hold significant potential for addressing future protein supply and demand. These proteins are derived from alternative and often more sustainable sources, including mycoproteins, microalgae, seaweed, plant-based meat substitutes, insects, and cultured meat [3,4].

Seaweeds are increasingly recognized by consumers for their health benefits due to their rich nutrient content, and their cultivation is considered more environmentally sustainable than traditional terrestrial crops [5,6]. Seaweeds are a promising source of protein, with numerous studies highlighting their significant levels of protein and essential amino acids (EEAs) [7]. Typically, the protein content of seaweeds ranges from 5% to 47% of their dry weight, varying among different types—red seaweeds constitute 10% to 30%, brown seaweeds constitute 5% to 15%, and green seaweeds constitute 3% to 47%. However, this composition can fluctuate depending on the species and cultivation conditions [8,9,10]. As such, seaweed is a promising source of protein with numerous recognized health benefits [11]. Seaweed proteins are theoretically challenging to digest due to the complex structure of their cell walls [12], which comprise water-insoluble cellulose microfibrils and water-soluble polysaccharides like xylans and alginates [13]. Despite these challenges, seaweeds remain a sustainable and healthy alternative as they contain all essential amino acids (EAAs) required in the human diet [14]. Although seaweeds are generally difficult to digest in their whole form, they remain a sustainable and healthy alternative because they contain all the essential amino acids (EAAs) required in the human diet [15]. Processing methods to concentrate seaweed proteins and enhance their digestibility have also been explored [16]. On the other hand, the protein quality of seaweed, particularly its essential amino acid (EAA) content and digestibility or bioaccessibility, has not been rigorously investigated via industry-standard methods. This is especially true for edible species found along the Chilean coasts that could be used in food applications. While metrics such as the protein digestibility-corrected amino acid score (PDCAAS) or the digestible indispensable amino acid score (DIAAS) cannot be accurately calculated because of the lack of in vivo protein digestibility evaluations, existing data on EAA content and in vitro digestibility could be used for estimation. This would provide a valuable comparison of seaweed proteins with traditional protein sources [7].

According to the Food and Agriculture Organization of the United Nations (FAO), true ileal digestibility (TID) measures the difference between the amino acids ingested and those recovered from the ileal digesta, adjusted for basal and specific endogenous amino acid losses [17]. While TID is commonly reported to be lower for plant-based proteins than for animal-based sources, this metric is essential for evaluating the bioavailability and nutritional quality of proteins, including those derived from novel sources such as seaweeds and mycoproteins [4,7,18]. Although mycoproteins have been produced using various substrates, such as agricultural by-products and industrial residues, research has predominantly focused on the amino acid composition and concentration of these proteins [19,20]. The majority of existing studies focus on the commercially available mycoprotein produced by *Fusarium venenatum* (marketed by Quorn), with limited research exploring the in vitro digestibility or protein digestibility-corrected amino acid scores (PDCAASs) of mycoproteins derived from alternative substrates [21].

In Chile, the intake of seaweed-based foods is relatively low despite the country’s extensive coastline, which is rich in edible seaweed. According to a 2019 report by the Undersecretariat for Fisheries and Aquaculture (SUBPESCA), only 0.19 kg of the 15 kg of seafood consumed per person annually consists of seaweed, with *Durvillaea antarctica* (“Cochayuyo”) being one of the few species regularly consumed [22]. This limited consumption is attributed mainly to a lack of awareness regarding its nutritional benefits, unfavorable sensory properties, and limited availability of seaweed-based products within the food industry [23]. Chile is also a major global seaweed producer, with *Macrocystis pyrifera* being one of the most extensively harvested species; it is used mainly for abalone feed and alginate extraction. Recently, new opportunities have emerged for *Macrocystis*, including its application in human food and the production of biofuels or chemicals, which could increase demand and promote the development of commercial cultivation systems. [24]. Another seaweed with significant potential for food and industrial uses, owing to its abundant biomass and nutritional value, is *Ulva* spp. (green seaweed). While *Ulva* spp. is not consumed in Chile, it is commonly consumed in dry form in coastal regions of China. [25]. Additionally, blooms of green algae from the *Ulva* genus have been persistent along the central Chilean coast, posing environmental challenges but also presenting opportunities for biomass utilization in food applications [26].

The aim of this study was thus to evaluate the digestibility of amino acids present in two brown seaweeds, *Durvillaea* spp. and *Macrocystis pyrifera*, which are widely used in the food industry for alginate extraction but could also represent a potential source of dietary proteins. Furthermore, this study assessed a green seaweed, *Ulva* spp., recognized and known for its nutritional value and use in food applications. A key objective was to compare the digestibility of a novel mycoprotein produced through the fermentation-based bioconversion of *Durvillaea* spp. by fungi with that of unfermented seaweed, demonstrating the potential improvement in protein quality through fermentation processes.

## 2. Results and Discussion

### 2.1. Protein Content and Amino Acid Composition

Among all the seaweed proteins evaluated in this study, seaweed-derived mycoproteins presented the highest protein content, with a value of 31.3% on a dry weight (DW) basis. It was followed by *Ulva* spp., *M. pyrifera,* and *Durvillaea* spp., which contained approximately 20.9%, 12%, and 11.8% protein on a DW basis, respectively (Table 1). Both brown seaweed samples (*Durvillaea* spp. and *M. pyrifera*) presented comparable protein contents, whereas *Ulva* spp. presented a 44% increase. Additionally, when processed and fermented into a mycoprotein, *Durvillaea* spp. presented a 61% increase in protein content. These protein levels are consistent with reports from other types of algae, where red algae typically present the highest protein concentrations (20–47% dw), followed by green algae (9–26% dw) and brown algae (3–15% dw) [27]. The protein content reported for mycoproteins derived from lignocellulosic biomass ranges between 40% and 50% (DW) [18]. This protein content is comparable to that of conventional legumes widely used in food, including peas, lentils, lupines, chickpeas, soybeans, fava beans, mung beans, and various other beans, which generally provide 20–40% dietary protein on a dry weight basis [28].

Regarding the total amino acids (AAs) in the crude protein, as shown in Table 1, both brown seaweed species presented similar concentrations, ranging from 84.3% to 90.1%. In contrast, the total AA content in the green seaweed *Ulva* spp. (65.7%) was significantly lower (*p* < 0.05) than that in the brown seaweed. For the seaweed-derived mycoproteins, the total AA content reached 74.7%, which was significantly different (*p* < 0.05) from that of the seaweed samples. The percentage of essential amino acids (EAAs) was similar between the seaweed-derived mycoprotein (39.3%) and *Durvillaea* spp. (38.3%). Moreover, *Ulva* spp. had an EAA proportion of 35.2%, and *M. pyrifera* had an EAA proportion of 31.4%. Notably, the EAA proportion in *Durvillaea* spp. was unaffected by bioprocessing, remaining consistent in both the raw seaweed and the seaweed-derived protein. Overall, all the samples presented a comparable percentage of EAAs, which aligns with values reported in previous studies on seaweed [29]. Conversely, these samples presented percentages of essential amino acids (EAAs) comparable to those of various plant-based protein sources, including soy (27%), brown rice (28%), pea (30%), corn (32%), and potato (37%). In contrast, animal-based proteins typically contain the highest EAA content, averaging approximately 43% [30]. The amino acid (AA) profiles of the samples are presented in Table 1. Among the most abundant essential amino acids (EAAs) identified in the mycoprotein and seaweed species, leucine was prominent, comprising 5.5% of the total amino acids in the seaweed-derived mycoprotein and 4.2%, 4.5%, and 5.8% in *Ulva* spp., *M. pyrifera*, and *Durvillaea* spp., respectively. Lysine was the second most abundant AA in the mycoprotein and *M. pyrifera*, at approximately 5%. In *Ulva* spp., however, the second most abundant AA was valine (3.9%).

Histidine was the least abundant AA in the seaweed samples, ranging from 1.0% to 1.72%, whereas methionine was the least abundant AA in the mycoproteins, at 1.7%. Threonine was not significantly different among the studied samples. The tyrosine content was notably similar in the mycoprotein (2.1%) and brown seaweed samples, while its concentration was significantly greater (*p* < 0.05) in the *Ulva* spp. green seaweed samples. Another abundant AA in these samples was phenylalanine, accounting for 4.3% and 5.0% of the brown seaweeds *M. pyrifera* and *Durvillaea* spp., respectively, whereas the mycoprotein and *Ulva* spp. had an approximate phenylalanine content of 3.5%.

The EAA composition observed in the green and brown seaweeds is consistent with values reported in other studies on marine algae [27]. In the case of other types of mycoproteins, such as *Pleurotus ostreatus* mycelium, which uses glucose and xylose as carbon sources, higher concentrations of essential amino acids (EAAs) were reported than in our study, with increases of up to 100% in valine, methionine, leucine, phenylalanine, and isoleucine. However, our study revealed higher values of tyrosine and lysine residues [31]. The essential amino acid (EAA) composition of commercial Quorn mycoprotein is lower than the EAA values reported in this study [32]. The essential amino acid (EAA) criterion is used to assess the amino acid profile of a protein source. As complete proteins, mycoproteins and many seaweed species contain all the EAAs, with some present in higher concentrations than others, emphasizing their nutritional superiority. The amino acid compositions of mycoprotein and seaweed were found to meet or exceed the EAA criteria established by the Food and Agriculture Organization (FAO) and the World Health Organization (WHO) [15,33,34]. Studies consistently report that methionine and cysteine are typically found at low concentrations in both seaweed and mycoprotein [20,35]. In this study, cysteine was undetectable, which aligns with previous findings where it has been reported at minimal levels [15]. Additionally, tryptophan and lysine are often identified as limiting amino acids in most algal species [35,36]. Leucine and isoleucine are commonly found at low concentrations in red algal species, whereas methionine, cysteine, and lysine are often identified as the limiting amino acids in brown algal species [35,36].

The content of nonessential amino acids (NEAAs) in crude protein (Table 1) was significantly greater (*p* < 0.05) in *Ulva* spp. (65.35%) than in the other samples. In contrast, the NEAA contents of brown algae and seaweed-derived mycoproteins were not significantly different, ranging from 53% to 59%. The content of glutamic acid notably increased in both the seaweed-derived mycoprotein and *Durvillaea* spp. at 14.4% and 19.9%, respectively. Moreover, glycine and proline prominently increased in *Ulva* spp. The seaweed-derived mycoprotein presented higher concentrations of arginine (5.6%), whereas *Durvillaea* spp. presented the highest alanine levels (7.5%).

Aspartic acid and glutamic acid represent a significant proportion of the total amino acids in numerous seaweed and fungal species, playing a key role in contributing to the characteristic ‘umami’ flavor commonly associated with seaweed [36,37]. For example, glutamic acid and aspartic acid have been shown to constitute 22–44% of the total amino acids in Fucus species and 26–32% in *Ulva* species [38]. Similarly, in fungi, the intracellular amino acid pool is predominantly composed of glutamate, aspartic acid, and alanine, with relatively small proportions of arginine, lysine, and histidine [39]. These amino acids, particularly glutamic acid and aspartic acid, are significant contributors to umami flavor and present potential as natural flavor enhancers, as evidenced in various fungal studies [40].

The EAA-to-NEEA ratio, ranging from 0.54 to 0.65, highlights the balance of essential and nonessential amino acids in the analyzed protein samples. These findings demonstrate that EAAs were consistently less abundant than NEAAs in all the evaluated samples. The highest ratio (0.65) was recorded for the seaweed-derived mycoprotein, followed closely by the brown seaweed *Durvillaea* spp. (0.62). Additionally, the EAA/NEAA ratios observed in other seaweed types align with previously reported values in the literature, falling within the range of 0.5–0.7 [35,41].

The quality of proteins for human and animal nutrition is primarily determined by their digestibility and essential amino acid (EAA) content. Although animal proteins are considered complete owing to their high EAA levels, their consumption should be moderated because of associations with cardiovascular diseases and diabetes [15,42]. Consequently, proteins from seaweed and mycoproteins offer an alternative that, when paired with other dietary protein sources, can provide a well-balanced and high-quality protein intake [18,27].

### 2.2. In Vitro Protein Digestibility, PDCAAS, and DIAAS Assessment

In vitro gastrointestinal digestion of the seaweeds and seaweed-derived mycoprotein was carried out via the enzyme digestion method provided by the Megazyme assay kit. Table 2 presents the essential amino acid content (mg/100 mg of crude protein) and amino acid scores for each sample, which were calculated based on the reference patterns for 1- to 2-year-old children, as outlined in [43]. The EAA score is a critical parameter for assessing protein quality, and a reference amino acid from a standard protein is used as a benchmark [42,43]. Among the samples, the seaweed-derived mycoproteins, followed by *Durvillaea* spp. and *M. pyrifera*, presented the best overall amino acid composition, with most essential amino acid scores reaching or approaching 1.0 in their respective patterns.

Limiting essential amino acids (EAAs) are key indicators of the nutritional value of algal proteins. In green seaweed (*Ulva* spp.), lysine (score of 0.6) and histidine (score of 0.6) were identified as the limiting EAAs. For the brown seaweeds *M. pyrifera* and *Durvillaea* spp., histidine was the limiting EAA (scores of 0.6 and 0.7, respectively). In contrast, the seaweed-derived mycoprotein had leucine (Score 0.8) as its limiting EAA. Previous studies have reported that lysine and histidine are commonly the limiting EAAs in various seaweed species [29,35]. On the other hand, in studies of mycoproteins, leucine has been reported as the most abundant amino acid, followed by threonine [20]. This partially aligns with our findings, where leucine was the second most abundant EAA, with a concentration of 55 mg/100 mg crude protein. However, compared with the standard amino acid scoring pattern established by the 2007 WHO/FAO/UNO report [43], leucine was identified in this research as the limiting EAA in the seaweed-derived mycoprotein.

The in vitro digestibility and PDCAAS values are shown in Table 3. The seaweed-derived mycoprotein had a significantly greater PDCAAS score (0.89 ± 0.08, *p* < 0.05) than *Durvillaea* spp. (0.53 ± 0.08), *Ulva* spp. (0.41 ± 0.02), and *Macrocystis pyrifera* (0.37 ± 0.05). The amino acid score combined with protein digestibility is a widely used method to determine the completeness of proteins for human consumption [43]. The PDCAAS is considered one of the most reliable methods for evaluating protein quality, accounting for both amino acid composition and digestibility. Scores range from 0 to 1.0, with 1.0 representing high-quality protein [43,44].

The seaweed-derived mycoprotein also exhibited significantly greater in vitro digestibility and an OPA score (~1.01). This suggests that the bioconversion process likely enhances protein digestibility by breaking down structural barriers or mitigating anti-nutritional compounds. Its PDCAAS (~0.89) aligns closely with the reported nutritional quality of traditional mycoproteins. While slightly lower than the near-perfect score of 0.996, this difference may be attributed to variations in amino acid profiles or residual anti-nutritional factors from seaweed components despite the bioconversion process [45,46]. Therefore, mycoproteins provide exceptional protein quality, supporting muscle effectively supporting muscle synthesis and meeting dietary protein requirements. Studies with human ileostomy patients have demonstrated that its PDCAAS score is nearly equivalent to the ideal scores of eggs and milk and even exceeds those of chicken and beef [20,47].

Significant differences emerged when the digestibility of seaweed-derived mycoprotein was compared with that of edible filamentous fungi. The digestibility of fungal proteins, measured as the degree of hydrolysis (DH%), ranges from 43% to 72% after gastrointestinal digestion, with particularly low values for *Fusarium venenatum* (14% in the gastric phase and 43.5% in the intestinal phase). These results align with studies suggesting that the proteolysis of fungal proteins is driven by the diffusion of digestive enzymes through their cell walls, which can limit hydrolysis compared with muscle proteins, which lack such structural barriers [46,48].

In contrast, seaweed-derived mycoprotein has a much greater digestibility, with a normalized value close to 1. This superior result may be attributed not only to the bioconversion process, which likely enhances enzyme accessibility by breaking down structural barriers, but also to the use of a different method for measuring digestibility. Specifically, a Megazyme enzymatic kit modified with the OPA method was utilized, providing a more precise protein digestibility assessment than traditional methods. These methodological differences, combined with the optimized bioconversion process, position this mycoprotein as a good protein source with digestibility levels approaching those of high-quality animal proteins (59–67%) [48,49]. This highlights the potential of fermentation or other processing techniques to increase digestibility and overall protein quality in seaweed-based foods.

The results for *Macrocystis pyrifera* and *Durvillaea* spp. are consistent with the moderate PDCAAS values reported for other brown seaweeds, such as *Fucus serratus* (0.63 ± 0.084) and *Alaria esculenta* (0.59 ± 0.021), suggesting that these species face similar protein digestibility challenges [10]. In this study, both algae presented low PDCAASs, reflecting deficiencies in essential amino acids, a trend also noted in prior research. The literature findings indicate moderate digestibility for brown seaweed *Undaria pinnatifida* (48%) and red seaweed *Palmaria palmata* (56%), likely due to high fiber content or other limiting compounds. While this study does not directly include red seaweeds, the digestibility values observed for *Macrocystis pyrifera* (47%) and *Durvillaea* spp. (55%) align with those reported for red and brown algae, suggesting shared structural and compositional challenges [27].

Compared with brown seaweed, the PDCAAS of *Ulva* spp. was lower, primarily because histidine is the limiting amino acid, despite its greater digestibility (0.52). This observation aligns with the literature on *Ulva* protein digestibility, which attributes reduced digestibility to a high carbohydrate content (65%), including fibers such as ulvan and cellulose, which increase viscosity and hinder enzyme access due to intact cell walls [50]. Additionally, phenolic compounds further contribute to reduced digestibility by forming complexes with proteins, although their impact can be mitigated by adding antioxidants during extraction to increase protein solubility and digestibility [51,52]. Compared with other plant-based protein sources, such as pea protein concentrate (80%), *Moringa oleifera* seed protein (81–89%), pigeon peas (96%), black beans (82%), and peanuts (96%) [29,53,54], the seaweed species in this study presented significantly lower digestibility. These findings underscore the need to develop strategies to improve the bioaccessibility of seaweed proteins. One promising solution is the production of seaweed-derived mycoprotein, which demonstrates the potential of fermentation and other processing techniques to substantially increase both digestibility and overall protein quality in seaweed-based foods.

The digestible indispensable amino acid score (DIAAS) is a method recently developed by the FAO and WHO to assess dietary protein quality on the basis of the digestibility of each essential amino acid at the ileum (end of the small intestine) [55]. The DIAAS is currently recognized as the most accurate method for routinely evaluating the amino acid quality of single-source proteins. A DIAAS value of 1.0 corresponds to 100%, indicating that the protein source fully meets the amino acid requirements for a specific reference population [56]. The DIAAS values, calculated via in vitro amino acid digestibility assay for the seaweed-derived mycoprotein and *Ulva* samples, are presented in Table 4. The calculation of DIAAS for brown seaweed samples was not feasible, likely because of limitations inherent in the Megazyme enzymatic digestion method combined with the amino acid profiling approach using HPLC with precolumn derivatization. Brown seaweeds contain complex oligosaccharides, such as alginate, fucoidan, and laminarin, which can interact with proteins and peptides. These polysaccharides may also interfere with the derivatization process or coelute with amino acids during chromatographic separation, complicating their detection and quantification [57].

Significant differences were observed in the DIAAS values for threonine (0.43), valine (0.18), lysine (0.59), and histidine (0.67) from the seaweed-derived mycoprotein and compared with *Ulva* spp., with the mycoprotein consistently having higher scores. An exception was noted for the combined tyrosine and phenylalanine score, which was greater in *Ulva* (0.28) than in the seaweed-derived mycoprotein (0.16). The DIAAS values are reported as minimum and maximum ranges (based on amino acid content), with the mycoprotein content ranging from 0.12 to 0.68 and the *Ulva* content ranging from 0.10 to 0.55. The results of this study on the digestibility of seaweed-derived mycoprotein and *Ulva* can be contextualized by comparing them with data from plant-based protein sources such as faba beans and raw pea flour. The DIAAS values for raw faba bean flour, which range from 0.13–0.16 (minimum) to 0.32–0.38 (maximum), emphasize the importance of considering both free and total amino acids to reflect protein quality accurately. This approach accounts for key factors such as digestion extent, bioavailability, and amino acid absorption. Seaweed-derived mycoproteins, with DIAAS values ranging from 0.12 to 0.68, present a wider range but generally lower scores than faba bean varieties such as Malik. However, this discrepancy can be attributed to differences in protein structure, hydrophobicity, charge, and food matrix composition [58,59].

### 2.3. Characterization of the Digestion Products

**Figure 1:**
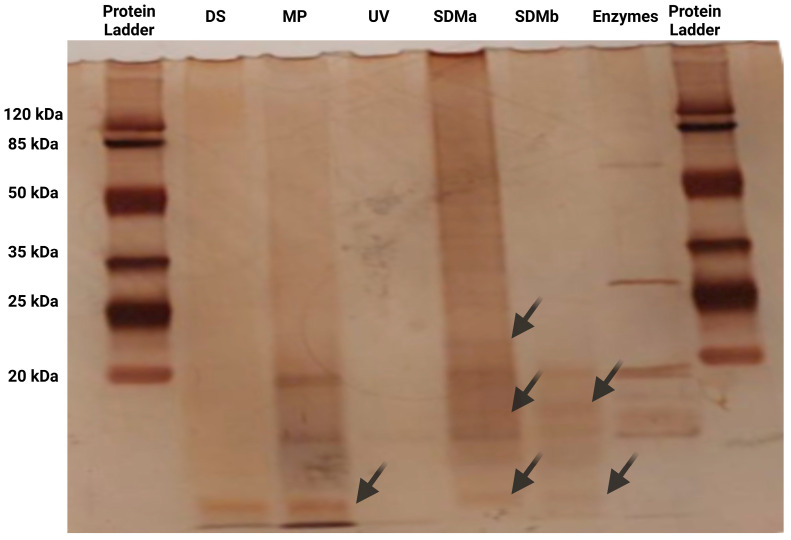
SDS-PAGE protein profiles of in vitro digested *Durvillaea* spp. (DS), *Macrocystis pyrifera* (MP), *Ulva* spp. (UV), and duplicates of seaweed-derived mycoprotein (SDMa and SDMb); enzymes: pepsin, trypsin, and chymotrypsin present in the digestibility assay kit Megazyme. The arrows indicate the peptides identified in the samples after digestion. These peptides are distinct from the enzymes used in the digestion assay.

Protein degradation during in vitro digestion and the potential presence of higher-molecular-weight proteins or peptide fragments after the intestinal phase were analyzed via SDS-PAGE, as shown in Figure 1. The seaweed-derived mycoprotein (SDM) samples subjected to intestinal digestion were run in duplicate (SDMa and SDMb). To improve the visualization of the bands, the samples were concentrated via ultrafiltration via Amicon filters with 30 kDa membranes. This step was necessary because untreated supernatants without ultrafiltration displayed intense, smeared staining with no clear bands visible. The analysis revealed the presence of a band below 25 kDa and two distinct bands below 20 kDa, which likely corresponded to soluble peptides generated through enzymatic hydrolysis facilitated by the fermentation process. The bands observed in the SDM samples result from the in vitro digestibility process, which reflects the enzymatic activity of the components in the digestibility kit used (Megazyme). This kit contains enzymes such as pepsin, trypsin, and chymotrypsin, which hydrolyze proteins into smaller fragments, resulting in the formation of peptides and smaller protein units. These hydrolysis products are visible as distinct bands in the samples, consistent with the expected outcomes of protein digestion under the specific conditions provided by the enzymatic assay. The smaller peptides identified in the mycoprotein samples may have significant nutritional and functional implications. Their reduced molecular size is likely to enhance absorption across the gastrointestinal tract and improve bioavailability. These bioactive peptides have been previously associated with health benefits, including antioxidant, antihypertensive, and immune-modulating activities. Similar results have been reported in other fermented products rich in bioactive peptides, supporting the potential of microbial fermentation as an innovative approach for producing peptides with health-promoting properties [60,61].

Among the digestion products of *Macrocystis pyrifera* and *Durvillaea* spp., a prominent band below 20 kDa was observed, suggesting the presence of specific proteins or peptides that remained partially hydrolyzed but soluble after enzymatic digestion. This finding indicates that the proteins in these seaweed samples possess a structure that is more accessible to enzymatic hydrolysis than those in other seaweed samples, likely due to their intrinsic protein composition and structural characteristics. In contrast, the digestion products of *Ulva* spp. did not display any detectable bands, indicating the absence of soluble peptides or proteins. This suggests that the proteins in *Ulva* spp. are likely bound to polysaccharides or form aggregates, potentially induced by thermal treatments applied during processing. Previous studies have reported that seaweed-derived hydrocolloids released during thermal processing can form gels through heating and cooling cycles. This gelation reduces protein solubility and complicates the analysis of digestion products. These differences in protein solubility and hydrolysis among the seaweed samples highlight the unique structural and biochemical properties of seaweed-derived proteins. Moreover, they underscore the impact of processing conditions on protein functionality and accessibility during digestion, emphasizing the need for optimized methods to preserve or increase protein bioavailability in seaweed-based products [62,63]. These structural changes support the hypothesis that processing induces significant alterations in seaweed protein structure, leading to the formation of aggregates that remain in the nonabsorbable fraction [29].

These findings highlight the ability of fermentation to produce soluble peptides in seaweed-derived mycoprotein, making fermentation a promising strategy for enhancing digestibility and functional properties. However, seaweed proteins present challenges related to their solubility and bioaccessibility, suggesting the need for further optimization to improve their nutritional potential.

## 3. Materials and Methods

### 3.1. Seaweeds and Seaweed-Derived Products

Herbamar™ (Concepción, Chile) supplied the seaweed *Durvillaea* spp. *Macrocystis pyrifera* was collected in October 2023 in Puerto Montt, Chile, and kindly provided by Dr. Buschmann (Universidad de Los Lagos). *Ulva* spp. was harvested in December 2023 in Coquimbo, Chile. The seaweed samples were dried at 40 °C, ground, and sieved to a particle size of 0.22 mm. The seaweed-derived product is an alternative protein supplied by Mycoseaweed^®^ Company (Santiago, Chile). Mycoseaweed^®^ has developed a technique to produce mycoprotein, an alternative protein from mycelia, via consortia (co-cultures) developed with different filamentous fungi and with *Durvillaea* spp. as the sole carbon source [16].

### 3.2. Analytical Procedures

#### 3.2.1. Total Protein Content and Analysis of Amino Acids

The total protein content in the samples was quantified via the Kjeldahl method, with a conversion factor of 6.25 applied to convert the nitrogen content to protein. The amino acid profiles of the three seaweed and seaweed-derived mycoprotein samples were determined via liquid-phase acid hydrolysis, as described by Landeta et al. (2024) [16]. The amino acid analysis was performed via an AccQ·Fluor Reagent Kit (WAT052880, Waters Corporation, Milford, MA, USA) on an HPLC system with a fluorescence detector (Shimadzu, Kyoto, Japan) and an AccQ-Tag Amino Acids C18 reversed-phase column (60 Å, 4 µm, 3.9 mm × 150 mm; Waters Corporation, Milford, MA, USA).

Mobile phase A consisted of 140 mM sodium acetate (AppliChem GmbH, Darmstadt, Germany), 20 mM triethylamine, (Merck KGaA, Darmstadt, Germany), and 3.42 mM (EDTA; AppliChem GmbH, Darmstadt, Germany) in water titrated to pH 5.02 with phosphoric acid (AppliChem GmbH, Darmstadt, Germany), while mobile phase B consisted of 60% acetonitrile (AppliChem GmbH, Darmstadt, Germany),in water (*v*/*v*). Quantification was conducted using an external amino acid standard H (NCI0180; Thermo Scientific™, Rockford, IL, USA).

#### 3.2.2. Protein Digestibility

The protein digestibility of the seaweeds and seaweed-derived mycoprotein was assessed using a protein or amino acid digestibility assay kit (Megazyme Ltd., Bray, Ireland) following the manufacturer’s protocol. Protein samples were digested sequentially as described previously [10] via pepsin and trypsin/chymotrypsin at neutral pH, and the undigested proteins were removed by precipitation with trichloroacetic acid (TCA; AppliChem GmbH, Darmstadt, Germany).

For the assay, 500 mg of each milled sample was used. To each sample, 19 mL of 0.06 M HCl (AppliChem GmbH, Darmstadt, Germany) was added, and the mixture was incubated at 37 °C with agitation at 150 rpm for 30 min. Then, 1 mL of pepsin solution was added to each sample, which was vortexed and further incubated for 1 h under the same conditions. Following pepsin digestion, the pH was adjusted to 7.4 with 2 mL of 1.0 M Tris buffer (Winker Ltd.a., Lampa, Santiago, Chile), pH 7.4. After thorough mixing, 200 µL of trypsin/chymotrypsin was added, and the samples were vortexed and incubated for 4 h at 37 °C with agitation at 150 rpm. The reaction was terminated by placing the samples in a boiling water bath for 10 min, followed by cooling to room temperature for 20 min. Subsequently, 1 mL of 40% TCA solution was added, and the samples were incubated overnight at 4 °C. After overnight incubation, the samples were centrifuged at 15,000 rpm for 10 min at room temperature. The resulting hydrolysates were stored at −80 °C until further analysis.

#### 3.2.3. Quantification of the Degree of Hydrolysis via the OPA Method

The degree of hydrolysis was evaluated based on the number of peptide bonds cleaved, with free amino groups quantified via the o-phthaldialdehyde (OPA; Sigma-Aldrich Chemie GmbH, Buchs, Switzerland) method described by Nielsen, Petersen, and Dambmann (2001) [64]. The OPA reagent was prepared by dissolving 160 mg of OPA in 4 mL of ethanol (J.T. Baker, Xalostoc, State of Mexico, Mexico) and mixing it with 150 mL of a solution containing 7.62 g of decahydrate sodium tetraborate and 200 mg of sodium dodecyl sulfate (Merck KGaA, Darmstadt, Germany). Subsequently, 176 mg of dithiothreitol (DTT, Winker Ltd.a., Lampa, Santiago, Chile) was added, and the volume was adjusted to 200 mL with deionized water. Serine Serine (0.9516 meqv/L; Merck KGaA, Darmstadt, Germany) was used as the standard.

For the assay, 100 µL of the sample or standard serine solution was mixed with 750 µL of the OPA reagent. The mixtures were incubated for 2 min at room temperature, and the absorbance was measured at 340 nm using a UV/VIS spectrophotometer (BMG LABTECH, Ortenberg, Germany).

#### 3.2.4. Characterization of Peptides from the In Vitro Simulated Digestion

The hydrolyzates were concentrated using 30 kDa and 10 kDa MWCO Amicon Ultra-15 membranes (Merck KGaA, Darmstadt, Germany), resulting in two concentrated fractions: 30 kDa and 10 kDa. The 10 kDa fraction was further analyzed by SDS-PAGE. Electrophoresis was performed with minor modifications following the protocol described in [29]. Samples were dissolved in a sample buffer containing Tris-HCl (0.05 M, pH 6.8), SDS (1.6% *w*/*v*), glycerol (8% *v*/*v*), β-mercaptoethanol (2% *v*/*v*), and bromophenol blue indicator (0.002% *w*/*v*). The samples were heated at 95 °C for 5 min and maintained at that temperature until loaded onto a 15% Bis-Tris polyacrylamide gel (AppliChem GmbH, Darmstadt, Germany). Electrophoretic separation was conducted at 140 V using a Mini-PROTEAN Tetra Cell (Bio-Rad Laboratories, Hercules, CA, USA). After separation, the gel was stained with silver nitrate for visualization.

### 3.3. Calculations of Nutritional Quality of the Protein

The nutritional quality of the proteins in the seaweeds and seaweed-derived mycoprotein was assessed by calculating the protein digestibility-corrected amino acid score (PDCAAS) and the digestible indispensable amino acid score (DIAAS). These are on the basis of the amino acid requirements for children aged 6 months to 3 years, as recommended by the Food and Agriculture Organization (FAO) of the United Nations [33].

#### 3.3.1. Protein Digestibility-Corrected Amino Acid Score (PDCAAS)

The PDCAAS was calculated via Equation (1) and considered the indispensable amino acid (IAA) [46]:

PDCAAS = 100 × (mg of limiting AA (1 g of sample) × digestibility (%))/(mg of the same AA in 1 g of the reference protein).(1)

#### 3.3.2. Digestible Indispensable Amino Acid Score (DIAAS)

The digestible indispensable amino acid ratio (DIAAR) was calculated for each indispensable amino acid (IAA) according to Equation (2). The digestible indispensable amino acid score (DIAAS) corresponds to the lowest DIAAR value obtained for each sample.

DIAAR = 100 × (mg of digestible dietary IAA (1 g of dietary protein)/(mg of the same IAA in 1 g of the reference protein))(2)

### 3.4. Statistical Analysis

The experiments were performed in triplicate, and the results are presented as the means ± standard deviations. One-way analysis of variance (ANOVA) was conducted using Statgraphics Centurion v.19 statistical software (Statpoint Technologies Inc., Warrenton, VA, USA). Differences were considered significant at *p* < 0.05 according to Tukey’s test.

## 4. Conclusions

This study highlights the potential of Chilean seaweeds, *Durvillaea* spp. and *Macrocystis pyrifera*, and seaweed-derived mycoproteins as alternative protein sources with promising applications in the food industry. Compared with the original seaweed biomass, the derived mycoprotein significantly increased the protein content (~33%), digestibility (~100%), and nutritional quality, emphasizing the effectiveness of bioconversion processes in improving protein bioavailability, while *Ulva* spp. exhibited greater digestibility (0.52) than brown seaweeds did, and its protein digestibility-corrected amino acid score (PDCAAS) was lower (0.37), indicating limitations in its amino acid balance. Nevertheless, the essential amino acid scores for *Ulva* spp. suggest a profile closer to the FAO reference pattern for some amino acids, particularly for methionine + cysteine (0.31) and histidine (0.54). In contrast, brown seaweeds presented lower digestibility and amino acid scores, likely due to structural barriers that limit accessibility during digestion. These findings highlight the importance of further characterizing the amino acid profiles of edible seaweeds and optimizing processing techniques, such as fermentation or bioconversion, to improve their digestibility and nutritional quality. Such efforts are crucial not only to develop high-quality, sustainable food ingredients that meet the dietary amino acid requirements set by the FAO but also to address the nutritional needs of a growing global population. Moreover, enhancing the nutritional value of seaweeds can play a vital role in improving food security and nutrition in Chile and other countries with access to seaweeds, offering a sustainable and healthy source of food.

## Figures and Tables

**Table 1:** Protein composition and amino acid profile of seaweed-derived mycoprotein, *Ulva* spp., *Macrocystis pyrifera* and *Durvillaea* spp.

Protein Composition	Seaweed-Derived Mycoprotein	*Ulva* Spp.	*Macrocystis* *pyrifera*	*Durvillaea* Spp.
Crude protein (g/100 g dw)	31.35 ± 3.69 ^c^	20.9 ± 1.79 ^b^	12 ± 1.61 ^a^	11.8 ± 1.17 ^a^
	Amino acid composition (g/100 g crude protein)			
Asp	7.24 ± 0.58	7.2 ± 0.19	9.02 ± 0.69	8.74 ± 0.18
Ser	3.61 ± 0.59 ^b^	5.96 ± 0.7 ^c^	2.34 ± 0.32 ^a^	2.87 ± 0.04 ^ab^
Glu	14.45 ± 0.93 ^c^	5.13 ± 0.23 ^a^	7.86 ± 0.61 ^b^	19.98 ± 0.44 ^d^
Gly	4.11 ± 0.52 ^ab^	6.37 ± 1.01 ^b^	2.74 ± 0.18 ^a^	3.62 ± 0.06 ^a^
His	1.96 ± 0.07 ^d^	1.0 ± 0.01 ^a^	1.56 ± 0.01 ^b^	1.72 ± 0.03 ^c^
Arg	5.59 ± 0.23 ^d^	3.44 ± 0.1 ^a^	2.93 ± 0.11 ^b^	4.21 ± 0.08 ^c^
Thr	3.94 ± 0.22	3.54 ± 0.14	4.21 ± 0.29	4.56 ± 0.06
Ala	5.07 ± 0.34 ^a^	5.49 ± 0.06 ^a^	4.8 ± 0.4 ^a^	7.53 ± 0.15 ^b^
Pro	3.21 ± 0.61 ^a^	5.85 ± 0.47	2.97 ± 0.65 ^b^	2.58 ± 0.01 ^a^
Tyr	2.12 ± 0.1 ^a^	3.11 ± 0.55 ^b^	2.36 ± 0.07 ^a^	2.48 ± 0.03 ^a^
Val	4.11 ± 0.27 ^ab^	3.96 ± 0.11 ^a^	4.03 ± 0.28 ^a^	4.87 ± 0.1 ^b^
Met	1.69 ± 0.08 ^d^	1.42 ± 0.03 ^a^	2.1 ± 0.06 ^c^	2.62 ± 0.1 ^a^
Lys	5.44 ± 0.52 ^c^	2.91 ± 0.05 ^a^	5.04 ± 0.44 ^bc^	4.28 ± 0.11 ^b^
Ile	3.14 ± 0.16 ^b^	2.54 ± 0.06 ^a^	2.44 ± 0.15 ^a^	3.47 ± 0.07 ^b^
Leu	5.5 ± 0.52 ^b^	4.28 ± 0.01 ^a^	4.57 ± 0.28 ^a^	5.81 ± 0.11 ^b^
Phe	3.61 ± 0.11 ^a^	3.54 ± 0.03 ^a^	4.38 ± 0.15 ^b^	5.04 ± 0.08 ^c^
∑ AA	74.78 ± 5.94 ^b^	65.74 ± 5.21 ^a^	80.12 ± 2.49 ^b^	84.37 ± 2.51 ^b^
% EAA	39.3 ± 2.15 ^c^	35.27 ± 3.09 ^b^	31.44 ± 1.18 ^a^	38.35 ± 1.72 ^c^
% NEAA	53.22 ± 2.07 ^a^	59.5 ± 3.89 ^b^	55.3 ± 6.44 ^c^	56.66 ± 3.34 ^ab^
EAA/AA	0.39 ± 0.04 ^b^	0.35 ± 0.03 ^b^	0.39 ± 0.02 ^a^	0.38 ± 0.01 ^b^
EAA/NEA	0.65 ± 0.05	0.54 ± 0.03	0.56 ± 0.04	0.62 ± 0.05

The data are expressed as the means ± standard deviations (SDs). a, b, c Different lowercase letters indicate statistically significant differences (*p* < 0.05) between the samples listed in the columns (seaweed-derived mycoprotein, *Ulva* spp., *Macrocystis pyrifera*, and *Durvillaea* spp.), analyzed individually for each row (i.e., for crude protein, each amino acid, and parameter: Σ AA, % EAA, % NEAA, EAA/AA, and EAA/NEAA). The crude protein content was determined via the Kjeldahl method.

**Table 2:** Indispensable amino acid composition and scores of seaweed-derived mycoprotein, *Ulva* spp., *Macrocystis pyrifera*, and *Durvillaea* spp.

Amino Acids	Pattern ^c^	Seaweed-Derived Mycoprotein	*Ulva* Spp.			*Macrocystis* *pyrifera*		*Durvillaea* Spp.	
		AA ^a^	Score ^b^	AA ^a^	Score ^b^	AA ^a^	Score ^b^	AA ^a^	Score ^b^
Thr	27	39.3 ± 2.2	1.0	35.3 ± 1.3	1.0	42.14 ± 2.93	1.0	45.59 ± 0.63	1.0
Val	42	41.1 ± 2.7	1.0	39.5 ± 1.1	0.9	40.29 ± 2.76	1.0	48.73 ± 1.0	1.0
Met + Cys	26	24.1 ± 0.7	0.9	46.15 ± 6.33	1.0	44.29 ± 0.56	1.0	26.19 ± 0.95	1.0
Ile	31	31.4 ± 1.0	1.0	25.35 ± 0.12	0.8	24.35 ± 1.45	0.8	34.6 ± 0.72	1.0
Leu	63	55.0 ± 0.9	0.8	42.79 ± 5.49	0.7	45.66 ± 2.81	0.7	58.07 ± 1.13	0.9
Tyr + Phe	46	57.2 ± 0.4	1.0	66.58 ± 0.33	1.0	67.45 ± 1.49	1.0	75.17 ± 0.83	1.0
Lys	52	54.4 ± 0.7	1.0	29.11 ± 0.49	0.6	50.40 ± 4.36	1.0	42.76 ± 1.12	0.8
His	18	19.5 ± 0.6	1.0	10.0 ± 0.12	0.6	11.56 ± 0.08	0.6	11.71 ± 0.27	0.7

^a^ AA = Amino acid composition (mg/100 mg crude protein); ^b^ Amino acid score = amino acid content in test protein/amino acid content of reference pattern; ^c^ Pattern = Amino acid scoring patterns for 1–2-year-old children according to the 2007 WHO/FAO/UNU report [43].

**Table 3:** In vitro digestibility and PDCAAS for seaweed-derived mycoprotein, *Ulva* spp., *Macrocystis pyrifera*, and *Durvillaea* spp.

Samples	In Vitro Digestibility OPA Method	PDCAAS
Seaweed-derived mycoprotein	1.01 ± 0.06 ^b^	0.89 ± 0.08 ^b^
*Ulva* spp.	0.52 ± 0.03 ^a^	0.37 ± 0.05 ^a^
*Macrocystis pyrifera*	0.47 ± 0.06 ^a^	0.41 ± 0.02 ^a^
*Durvillaea* spp.	0.55 ± 0.04 ^a^	0.53 ± 0.08 ^a^

The data are expressed as the means ± standard deviations (SDs). a, b Different lowercase letters indicate significant differences (*p* < 0.05) in the parameters evaluated. OPA, o-phthaldialdehyde; PDCAAS, protein digestibility-corrected amino acid score.

**Table 4:** DIAAS for seaweed-derived mycoprotein and *Ulva* spp.

Amino Acid	DIAAS	
	Seaweed-Derived Mycoprotein	*Ulva* Spp.
Thr	0.43 ± 0.02 ^b^	0.36 ± 0.03 ^a^
Val	0.18 ± 0.04 ^b^	0.14 ± 0.02 ^a^
Met + Cys	0.33 ± 0.02 ^a^	0.31 ± 0.04 ^a^
Ile	0.12 ± 0.04 ^a^	0.09 ± 0.02 ^a^
Leu	0.19 ± 0.03 ^a^	0.20 ± 0.03 ^a^
Tyr + Phe	0.16 ± 0.03 ^b^	0.28 ± 0.05 ^a^
Lys	0.59 ± 0.02 ^b^	0.17 ± 0.02 ^a^
His	0.67 ± 0.09 ^b^	0.54 ± 0.09 ^a^

The data are expressed as the means ± standard deviations (SDs). a, b Different lowercase letters indicate significant differences (*p* < 0.05) in the parameters evaluated. DIAAS, digestible indispensable amino acid score.

## Data Availability

The raw data supporting the conclusions of this article will be made available by the authors on request.
